# Incidence, Prevalence, and Mortality Trends in Chronic Obstructive Pulmonary Disease over 2001 to 2011: A Public Health Point of View of the Burden

**DOI:** 10.1155/2016/7518287

**Published:** 2016-07-11

**Authors:** Mariève Doucet, Louis Rochette, Denis Hamel

**Affiliations:** ^1^Department of Medicine, Laval University, Quebec, QC, Canada G1V 0A6; ^2^Institut national de santé publique du Québec, Quebec, QC, Canada G1V 5B3

## Abstract

*Background*. An increase of chronic obstructive pulmonary disease (COPD) prevalence was reported in Canada despite the decline of the main risk factor.* Objectives*. To estimate incidence, prevalence, and mortality of COPD from 2001 to 2011 and establish the COPD burden by the evaluation of the age-period-cohort effects on incidence trends and the comorbidities prevalence estimations.* Methods*. A retrospective population-based cohort was built using Quebec health administrative data. Change in trends was measured by relative percentage of changes and by joinpoint regression. After a descriptive analysis of the trends, an age-period-cohort analysis was performed on incidence rates.* Results*. Overall increase in prevalence along with a decrease of incidence and all-cause mortality was observed. Over time, all age-standardized trends were higher in men than women. Despite higher rates, the number of incident and prevalent cases in women exceeds men since 2004. The curve analysis by age groups showed over time a downshift for both sexes in incidence and all-cause mortality. Further analysis showed the presence of a cohort effect in women.* Conclusion*. The burden of COPD has risen over time. Women younger than 65 years old have been identified as at-risk group for healthcare planning.

## 1. Introduction

Chronic obstructive pulmonary disease (COPD) is a progressive disease associated with successive exacerbation episodes that lead to frequent hospitalization [1]. Furthermore, this respiratory disease is frequently associated with comorbid conditions that make COPD, a disease with heavier clinical management [2, 3]. Effort to estimate adequately the COPD burden was made internationally; the population-based study, Burden of Obstructive Pulmonary Disease (BOLD), has challenged previous results reported by population-based survey that obtained lower prevalence estimate with self-report diagnosis [4, 5]. This international study, based on self-report and measured data, has reported a prevalence of 10.1% in moderate to severe COPD worldwide (GOLD II or higher) [4].

The Canadian initiative of BOLD, the COLD study, has estimated that COPD afflicts more than 15.0% of Canadians in early stage (GOLD I or higher) and 7.9% in GOLD II or higher [6]. Another Canadian population-based study has instead used health administrative data to estimate the COPD burden and has observed an overall prevalence of 9.5% [7]. The same study has reported a substantial increase of the prevalence along with a decrease of incidence and all-cause mortally trends [7].

This rise of overall COPD prevalence is interesting although over 30 years of cigarette smoking prevalence declined, in addition to a marked decrease of mortality over the last decade. The link between incidence, prevalence, and mortality in COPD needs to be explored in order to draw a proper picture of its burden.

In the province of Quebec, the second province in population size in Canada, a surveillance system of chronic diseases based on an access to a linked health administrative database was used to estimate the burden of COPD [8, 9].

The main objective of this study was to estimate, for the period of 2001 to 2011, incidence, prevalence, and mortality of COPD individuals among 35 years and older using Quebec health administrative data.

Furthermore, this study will (1) estimate the overall trends in terms of sex and age groups as well as identify years with potential variation in the trend. In a second step this study will focus on the understanding of the prevalence trend in order to draw a proper picture of the COPD burden, (2) explore the potential effects of age, period, or cohort effect on incidence trends, and (3) describe, in COPD population, the prevalence of seven main comorbidities that are identified for public health surveillance.

## 2. Methods

### 2.1. Study Population

A retrospective population-based cohort was built using linked health administrative data used for surveillance of chronic diseases in Quebec [8]. This system represents almost the entire population of Quebec and includes (1) the health insurance registry of the* Régie de l'assurance maladie du Québec* (RAMQ) that contains sociodemographic information for nearly all of population of Quebec, (2) fee-for-service data (physician billing), (3) hospital discharge as well as (4) drug data for the 65 years and older, and (5) mortality data. The present study covers all residents insured with the Quebec universal health insurance plan recorded in the RAMQ database, from January 1, 1996, to March 31, 2012.

#### 2.1.1. COPD Cohort

Identification of diagnosed COPD case was defined as at least one visit to a physician, OR one hospitalization with a diagnosis of COPD from all available diagnostic fields, among individuals 35 years or older. COPD diagnoses were identified through ICD-9 codes 491-492 and 496 or ICD-10-CA J41–44. This validated definition case was associated with a sensitivity of 85% (95% CI: 77.0% to 91.0%) and a specificity of 78.4% (95% CI: 73.6% to 82.7%) [10].

A run-in period of five years was necessary [7]; therefore this study covered the period of April 1, 2001, to March 31, 2012.

### 2.2. Indicators

#### 2.2.1. Incidence Rate

The number of new annual cases of COPD is divided by the at-risk population.

#### 2.2.2. Prevalence

The number of diagnosed cases of COPD is divided by population.

#### 2.2.3. Prevalence of Comorbidities

Prevalence of comorbidities measured in individuals with and without COPD will be presented by sexes for the period 2011-12. Prevalence ratios of COPD on individuals without COPD will reflect excess of chronic diseases in COPD. Comorbidities measures represent prevalent chronic diseases [8, 11] that have been reported to be associated with COPD [2, 12] (Table 1).

#### 2.2.4. All-Cause Mortality Rate

The number of deaths due to any cause according to case status, with or without COPD, is divided by the population.

### 2.3. Statistical Analysis

All rates and rate ratios were age-standardized to 2001 Quebec population. The standardization procedure was done using the usual direct method.

#### 2.3.1. Trend Analysis

The overall change of the prevalence trend was measured by the relative percentage of changes. This method calculates the percentage of changes between the rates of two fiscal years using as the reference the rate of the earlier year, ([*T*_Final_ − *T*_Initial_]/*T*_Initial_) × 100.

Identification in the trend variation of incidence and all-cause mortality was performed by a regression analysis developed by the National Cancer Institute for trend analysis [13]. Joinpoint Regression is a software (version 4.0.4—May 2013; http://surveillance.cancer.gov/joinpoint/) that uses several different segments that are connected together at the “joinpoints,” takes trend data, and fits the simplest joinpoint model that the data allow. The annual percentage of change (APC) in rates with 95% CIs is provided for each segment between 2 “joinpoints,” and a permutation test was used to select the model that best fitted the data.

In addition, a statistical test for comparing age-standardized rates [14] was used to compare men with women in different age groups of age-specific incidence and in all-cause mortality for 2001 and 2011. The same method of comparisons was employed in the prevalence of each comorbidity and to identify significant differences between sexes in rate ratios.

#### 2.3.2. Age-Period-Cohort Analysis

This model considers three factors in the analysis [15]. The age factor that is associated with the physiological change in individual [15]. The period factor represents an external influence [15], such as a new governmental policy on smoking restriction. This factor influences almost all individuals at the same time. The birth cohort factor affects all individuals that are born within the same years [15], such as smoking habit in a generation.

To evaluate the effects of these interconnected variables, four periods of three years that extend from 2000–02 to 2009–11 were grouped together in 17 age groups (from 35–37 to 83–85) for 17 birth cohorts from 1922–24 to 1970–72.

A multiphase approach combining graphical inspection followed by the median Polish method (second-order effects are estimated and interpreted) and the Holford method (first-order effects are estimated with second-order effects interpreted) [16, 17]. A positive mean residue from the median Polish method suggests that the presence of a cohort effect with an observed rate is more superior than expected (without cohort effect).

## 3. Results

### 3.1. COPD Cohort

Based on the definition case used, 444,709 (males: 212,270, females: 232,432) individuals over 35 years old were identified with COPD in the province of Quebec, in 2011-12.

### 3.2. Incidence

**Figure 1:**
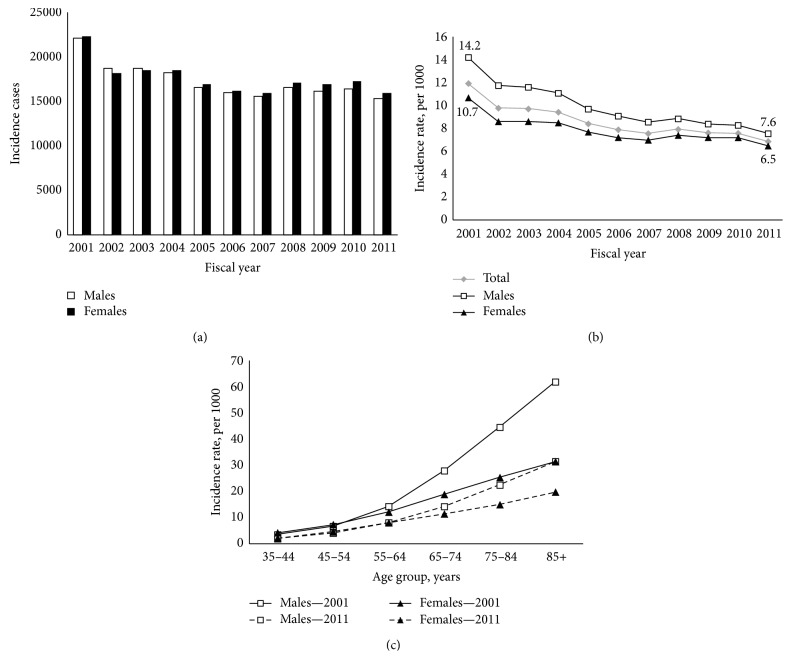
COPD incidence. (a) Number of incidence diagnosed COPD cases, 35 years and older, by sex, Quebec, 2001–2011. (b) Age-standardized COPD incidence rates, 35 years and older, by sex, Quebec, 2001–2011. (c) Age-specific COPD incidence rates, by sex, Quebec, 2001 compared to 2011 period.

In all diagnosed COPD aged 35 years and older, the number of newly diagnosed cases decreased from 2001 to 2011 (44,400 to 31,318) and this was true in men (22,107 to 15,363) and in women (22,293 to 15,955) (Figure 1(a)).

Over the period, age-standardized COPD incidence rates decreased from 12.0 to 6.9 per 1000 (Figure 1(b)). This observation was seen in both sexes (Table 2).

The incidence rate of individuals with COPD rose with increasing age. In 2011, the age-specific incidence rates were higher in men than in women beyond 65 years old (*p* ≤ 0.01). In contrast to 2011, the difference between sexes arrived at a younger age in 2001 (55 years and older, men compared to women, *p* ≤ 0.01) (Figure 1(c)).

### 3.3. Prevalence

**Figure 2:**
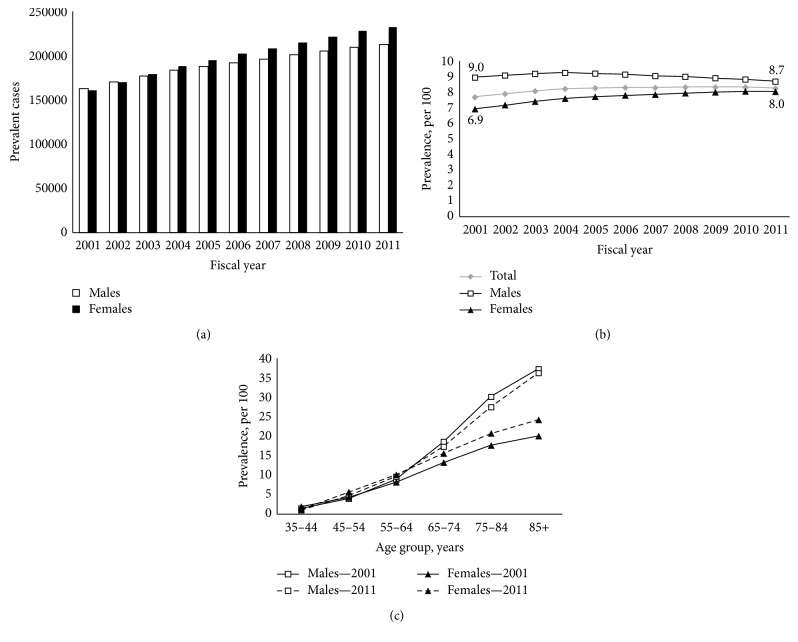
COPD prevalence. (a) Number of prevalent diagnosed COPD cases, 35 years and older, by sex, Quebec, 2001–2011. (b) Age-standardized COPD prevalence, 35 years and older, by sex, Quebec, 2001–2011. (c) Age-specific COPD prevalence, by sex, Quebec, 2001 compared to 2011 period.

The number of prevalent cases increased from 2001 (men: 163,128; women: 160,652) to 2011 (men: 212,270; women: 232,432) (Figure 2(a)) with an age-standardized prevalence that rose from 7.7% to 8.3% until 2011. This trend increase was seen in the first part of the period (2001 to 2004: relative increase of 6.9%) and then showed a plateau in the later part (2004 to 2011). Since 2004, a relative decrease of 6.0% in men as well as a relative increase of 5.8% was observed in women. For the overall period, age-standardized prevalence was higher in men compared to women, but this increase seems to be borne by women (Figure 2(b)).

Such as incidence rate, the age-specific prevalence increased with age. Similar patterns were observed between men and women despite a higher prevalence in men compared to women in 2011 (beyond 65 years old) and in 2001 (beyond 55 years old) (Figure 2(c)).

### 3.4. All-Cause Mortality

**Figure 3:**
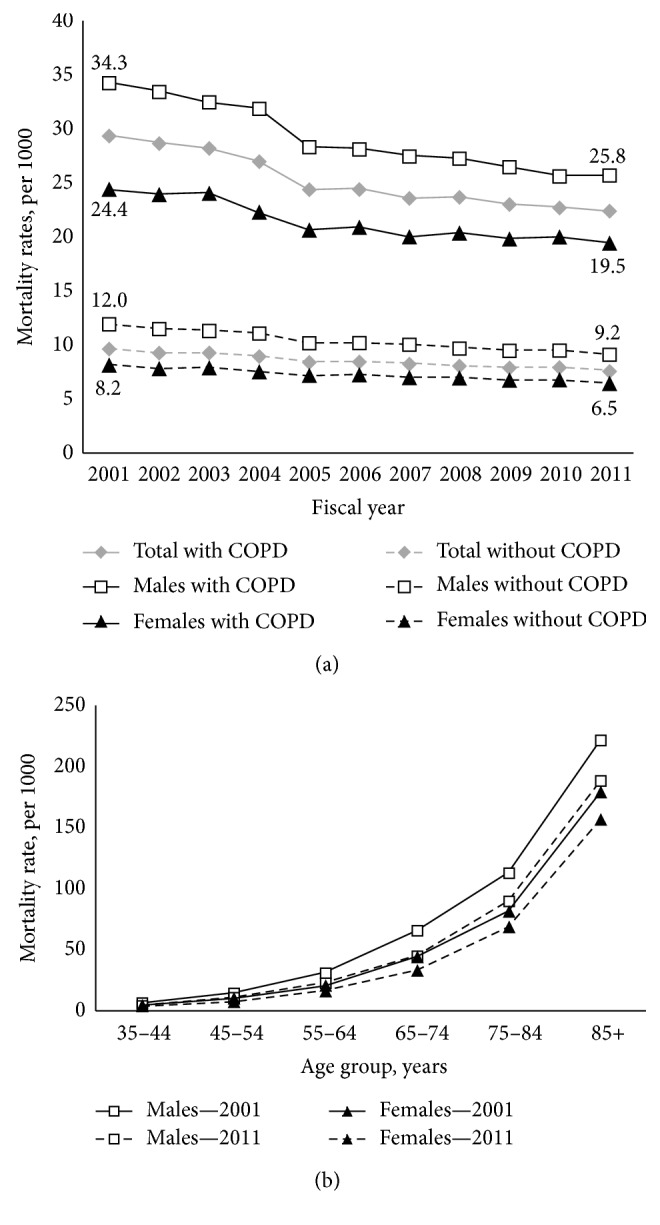
COPD all-cause mortality. (a) Age-standardized all-cause mortality rates among individuals aged 35 years and older with diagnosed COPD to those without diagnosed COPD, by sex, Quebec, 2001–2011. (b) Age-specific COPD all-cause mortality rates, by sex, Quebec, 2001 compared to 2011 period.

Overall, the age-standardized all-cause mortality rates decreased from 29.4 to 22.5 per 1000 in 2001 to 2011 (Figure 3(a)) and this decline was statistically significant in men and in women (Table 3). In addition, the all-cause mortality rates were higher in COPD compared to individual without COPD.

In 2011, the age-specific all-cause mortality rates beyond 45 years old were higher in men than in women in all age groups and this observation was also true for the period of 2001 (*p* ≤ 0.01) (Figure 3(b)).

### 3.5. Age-Period-Cohort Model for Incidence Trends

The graphical representations were not shown because the visual interpretation of the results was not convincing.

**Figure 4:**
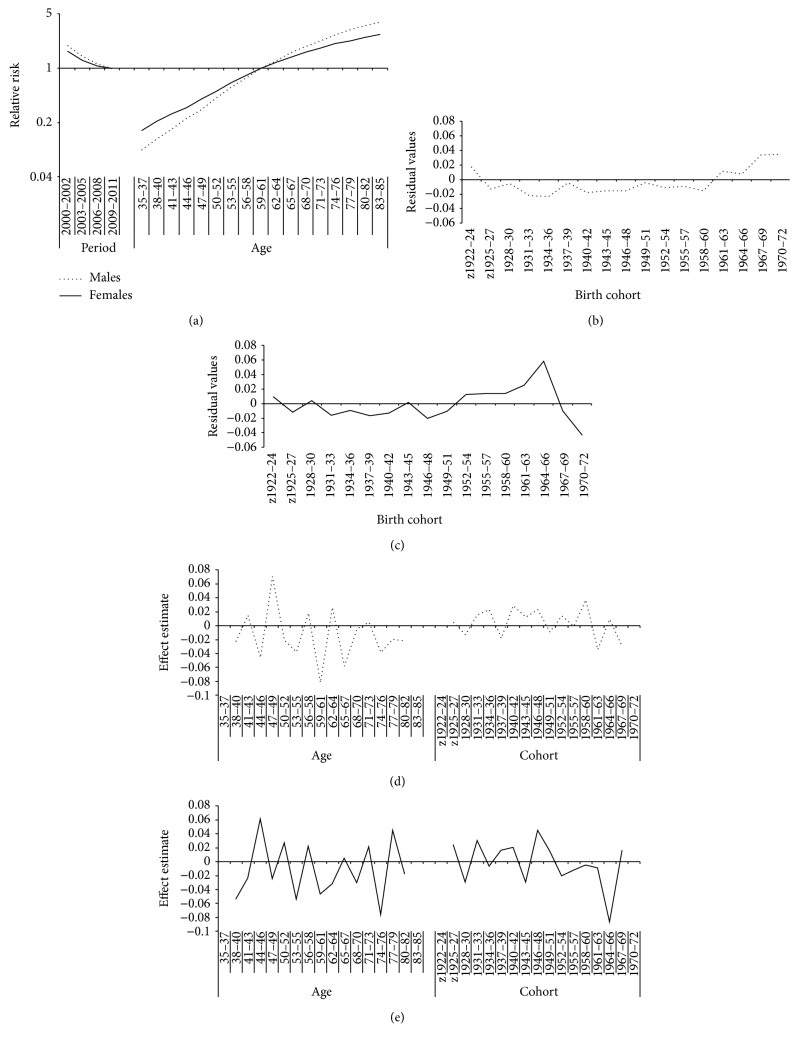
Age-period-cohort effects in COPD incidence rates for males and females. (a) Age and period effects, by sex. (b) Cohort effect in men (median Polish method). (c) Cohort effect in females (median Polish method). (d) Cohort effect in men (Holford method). (e) Cohort effect in females (Holford method).

The age and period effects by sex are presented in Figure 4(a). In both sexes, the risk of developing COPD was rising with age. The relative risk was higher in men compared to women in age groups of 65 years and older. For the period effect, a similar pattern between men and women was observed. The relative risk of developing the disease slowly diminished until the end of the observational period.

The cohort effect realized by the median Polish method analysis was presented for men in Figure 4(b) and for women in Figure 4(c). Residuals values obtained from median Polish method were similar in birth cohorts before 1949–51 in both sexes. A systematic deviation of residuals from zero was not statistically significant, suggesting a bit positive cohort effects in men in birth cohorts 1961–63 until 1970–72. A more important cohort effect was found in women by positive mean residuals in 1952–54 to 1967–69 birth cohorts. An increase to a significant peak of values in 1964–66 followed by a drop in 1967–69 that reach zero was observed.

In addition to the median Polish method, the Holford method (Figures 4(d) and 4(e)) confirms that the cohort effect seen in women was similar to the age effect. The important change in the second-order effects around 1964–66 suggests a cohort effect in women born during this specific period. Therefore, in men, the Holford method shows a less important cohort than age effect, but the variability observed in the recent cohorts seems to identify a weak cohort effect as found in median Polish method.

### 3.6. Prevalence of Comorbidities

**Figure 5:**
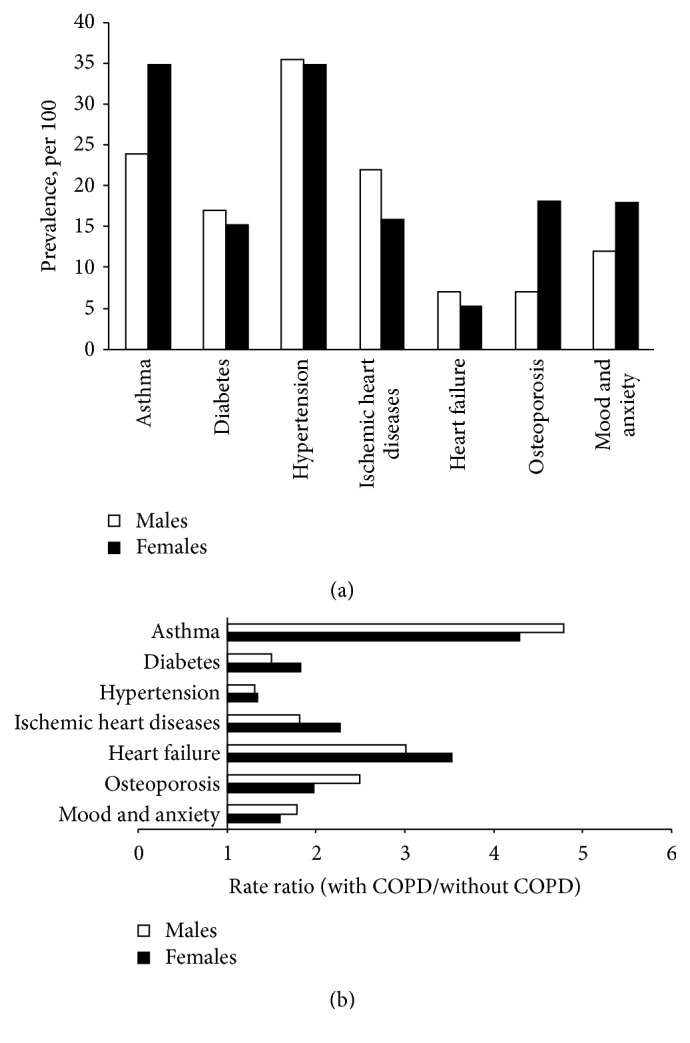
Comorbidities prevalence. (a) Age standardized comorbidities prevalence among COPD, 35 years and older, by sexes for the period 2011. (b) Rate ratios of age standardized comorbidities rate among individuals with and without COPD 35 years and older, by sexes for the period 2011.

Age-standardized prevalence of seven comorbidities in COPD and rate ratios for each chronic disease is presented by sexes in Figures 5(a) and 5(b), respectively. The co-occurrence of chronic diseases prevalence in both sexes was frequent in individuals with COPD. Asthma, osteoporosis, and mood and anxiety troubles were more prevalent in women with COPD (*p* ≤ 0.01) while diabetes, ischemic heart diseases, and heart failure were more prevalent in men with COPD (*p* ≤ 0.01). The co-occurrence of hypertension was equivalent in both sexes. Excess of co-occurrence of chronic diseases in individuals with COPD was found in both sexes. The prevalence ratios of diabetes, hypertension, ischemic heart diseases, and heart failure were more marked in women than in men (*p* ≤ 0.01).

## 4. Discussion

For the last decade, an overall increase in COPD prevalence along with a decrease of incidence and all-cause mortality was reported in Quebec. Over time, all age-standardized trends were higher in men compared to women. Despite higher rates, the number of incident and prevalent cases of COPD in women exceeds men since 2004. The curves analysis by age group showed in incidence and all-cause mortality a downshift for both sexes over time. This signified that all age groups presented lower rate in 2011. Further analysis on incidence trends shows the presence of a cohort effect in younger women. Altogether, these results support an increase of the burden of COPD that was mostly borne by women in the last decade.

### 4.1. Relationship between Incidence Prevalence and Mortality

The interrelation between incidence, prevalence, and mortality in COPD is complex by the presence of different pattern overtime between sexes. This relationship could be perceived in epidemiology as the representation of a bathtub with the measure of incidence representing the flow of water [18]. The reduction of COPD incidence rates over time suggests that combined efforts in public health to reduce cigarette smoking rates have begun to affect the number of new cases diagnoses with the disease. Over the past fifty years, the decrease of the prevalence of cigarette smoking is reported in North America [19]. In Canada, the peak of prevalence smoking in men was hypothetically identified before 1950 while in women the peak occurred around the mid-1960s [19]. The time gap between sexes in habit and cessation of cigarette smoking could explain the different pattern observed. Therefore, the number of incident cases in women exceeds men over time and the decline of rates in women was less marked in all age groups than in men in 2011. This observation may support a link between incidence and cigarette smoking patterns.

Secondly, the prevalence pool, figured by the amount of water fill in the bathtub, is also influenced by the outflow down the drain represented in this study by mortality data. Since cause of death by COPD is largely underestimated [12, 20] and trends are declining as well as all-cause mortality in COPD but at lower rate, the measure of all-cause of mortality was used to understand this relationship with prevalence. In this study, all-cause mortality trends decrease overtime and this was true in all age groups. In addition, the pattern of all-cause mortality was similar in men and in women.

### 4.2. Incidence in COPD Influence by a Period and Cohort Effect

Age-period-cohort analysis showed a period effect in both sexes. The relative risk decline suggests that COPD of different generations was all affected during the analyzed period. This observation could be explained by the impact of new tobacco control policies established in Canada in the 80s. Concurrently, the decline in other chronic diseases incidence with high risk factor related to cigarette smoking, such as lung cancer in men [21], could reinforce this hypothesis. Indeed, other factors such as the changes in medical practices or environmental factors may have also contributed but with less impact.

In addition to the period effect, a cohort effect was identified in women. The birth cohort factor affects individuals that are born within the same years. The analysis demonstrates an elevated relative risk of incidence in cohorts of women born between 1952 and 1967. Despite an overall decline of incidence trend in women in time, the diagnosis of COPD in women belonging to the most recent cohorts was higher. These results support previous observations made with our surveillance system that age-specific incidence and prevalence trends in women age beyond 55 years old were higher than men for the overall period. However, these findings in younger groups were not consistent with those in other age groups. High incidence and prevalence rates in younger women were also found in other countries [22, 23]. These authors explained their findings by different smoking habits between genders and between younger cohorts of COPD women. In Japanese COPD population, an age-period-cohort analysis performed on mortality rate observed also changes in cohort effect. They conclude that cigarette consumption and smoking prevalence variations over time explain differences in sexes [22].

### 4.3. Comorbidity Prevalence in COPD

Co-occurrence of chronic disease is now well recognized in the trajectory of the COPD disease. In women with COPD, asthma, osteoporosis, and mood and anxiety disorders were more prevalent. In addition, rate ratios showed an excess of heart failure, ischemic heart diseases, and diabetes in women than in men. In support to other studies [2, 3, 24], the presence of comorbidities was highly prevalent in COPD. Furthermore, women presented different pattern of comorbidity compared to men and these differences should be considered in the estimation of the burden.

### 4.4. Limitations

The definition case used for COPD surveillance contains limits. The modest specificity and positive predictive value of the health administrative data definition case lead to the presence of false positive. Despite a probable overestimate of the prevalence, the overall prevalence estimated in 2011 is consistent with prevalence found in other Canadian population-based data sources that identify the diagnoses of COPD based on spirometry measures [5, 6, 9]. Comparison between other sources of data has highlighted that our estimates may include predominantly moderate to severe COPD, which may underestimate the overall prevalence found with administrative data [9]. In this study, estimates measured are dependent on the diagnose record on physician billing and are an image of the clinical practice. Since COPD is underdiagnosed by physician, the true COPD prevalence in clinical practice is probably also underestimated. Consequently, estimates produced by administrative data give a population portrait of individuals who interact with healthcare system.

The age-period-cohort model leads to certain limits of interpretation such as the model itself that may not identify the cause of the effect observed. Finally, our relatively short observation period could have had limited the estimation of the burden and the analysis for age-period-cohort. However, all hypotheses put forward with this kind of analysis need to be confirmed in time.

## 5. Conclusions

Over the last decade, prevalence rates of diagnosed COPD increased while incidence and all-cause mortality rates decreased. The burden of COPD has risen over time and is mostly associated with women. An estimation of the last decade has identified women, younger than 65 years old, as an at-risk group. Public health and the health system should target this group in smoking cessation and in screening and managing the early stage of COPD disease in order to reduce the increase of COPD burden.

## Figures and Tables

**Table 1:** 

Comorbidities	ICD-9	ICD-10	Physician visit requirement	Hospital discharge requirement
Asthma	493	J45-46	2 visits or more within any 2-year period during study for asthma	1 discharge during study with asthma in any diagnostic field

Diabetes	250	E10–E14	2 visits or more within any 2-year period during study for diabetes	1 discharge during study with diabetes in any diagnostic field

Hypertension	401–405	I10–I13, I15	2 visits or more within any 2-year period during study for hypertension	1 discharge during study with hypertension in any diagnostic field

Chronic heart diseases	428	I50	2 visits or more within any 1-year period during study for chronic heart disease	1 discharge during study with chronic heart disease in any diagnostic field

Ischemic heart diseases	410–414	I20–I25	2 visits or more within any 1-year period during study for ischemic heart disease	1 discharge with any diagnostic field or 1 treatment code^1^ for ischemic heart disease during study

Osteoporosis	733	M80-M81	1 visit during the study for osteoporosis ever	1 discharge with osteoporosis in any diagnostic field ever

Mood or anxiety disorder	296, 300, 311	F30–F39, F40–F48, F68	1 visit during the study for mood disorder or anxiety disorder in one year (has to requalify every year)	1 discharge during the study for mood disorder or anxiety disorder in any diagnostic field in one year (has to requalify every year)

^1^CCADTC: 48.02, 48.03, 48.11–48.19/CCI: 1.IJ.50, 1.IJ.57.GQ, 1.IJ.54, 1.IJ.76.

**Table 2:** 

Incidence	Segment	Both sexes		Males		Females	
		APC (%)	95% CI	APC (%)	95% CI	APC (%)	95% CI
35+ years	2001–06	−6.9^*∗*^	−10.4 to −3.4	−7.8^*∗*^	−10.9 to −4.7	−3.4^*∗*^	−10.3 to −2.1
	2006–11	−1.8	−5.8 to 2.3	−2.9	−6.4 to 0.7	−1.1	−5.7 to 3.9

^*∗*^Joinpoint analysis shows statistically significant segment, *p* ≤ 0.05.

APC: annual percentage of change.

**Table 3:** 

Mortality	Segment	Both sexes		Males		Females	
		APC (%)	95% CI	APC (%)	95% CI	APC (%)	95% CI
With COPD (35+ years)	2001–11	−2.9^*∗*^	−3.5 to −2.3	−3.1^*∗*^	−3.7 to −2.6	−2.4^*∗*^	−3.1 to −1.7

^*∗*^Joinpoint analysis shows statistically significant segment, *p* ≤ 0.05.

APC: annual percentage of change.
